# 基于金属有机骨架材料复合气凝胶的分散固相萃取-超高效液相色谱-串联质谱法测定水中7种苯氧羧酸类除草剂

**DOI:** 10.3724/SP.J.1123.2024.01005

**Published:** 2024-11-08

**Authors:** Xin ZHANG, Gege WU, Wenlian CUI, Shuang LI, Jiping MA

**Affiliations:** 1.青岛理工大学环境与市政工程学院, 山东 青岛 266000; 1. College of Environmental and Municipal Engineering, Qingdao University of Technology, Qingdao 266000, China; 2.山东省青岛生态环境监测中心, 山东 青岛 266000; 2. Qingdao Ecological Environment Monitoring Center of Shandong Province, Qingdao 266000, China

**Keywords:** 超高效液相色谱-串联质谱, 分散固相萃取, 复合气凝胶, 金属有机骨架材料, 苯氧羧酸除草剂, 环境水体, ultra-high performance liquid chromatography-tandem mass spectrometry (UHPLC-MS/MS), dispersive solid phase extraction (DSPE), composite aerogel, metal-organic frameworks, phenoxy carboxylic acid herbicide, environmental waters

## Abstract

苯氧羧酸类除草剂(PCAs)在水体中难以降解,对人类健康和生态环境造成严重威胁,因此亟需建立有效测定水体中痕量PCAs的方法。本文以常温干燥法制备的金属有机骨架/海藻酸钠气凝胶材料MIL-101(Fe)-NH_2_/SA作为分散固相萃取的吸附剂,对环境水体中的7种PCAs进行吸附和富集,从萃取条件(气凝胶中MIL-101(Fe)-NH_2_与海藻酸钠比例、水样pH、萃取时间)、洗脱条件(洗脱溶剂比例、洗脱时间、洗脱体积)、离子强度(盐度)等方面对萃取效果进行优化,以获得最佳的萃取效果。优化结果显示,吸附剂在12 min内可以对目标物进行完全吸附,用总体积为4 mL的含1.5%甲酸的甲醇洗脱30 s,目标物可以充分解吸。在最优条件下,结合超高效液相色谱-串联质谱法(UHPLC-MS/MS),建立了基于金属有机骨架复合气凝胶测定水体中7种PCAs的新方法。该方法可以呈现良好的线性关系(*r*^2^≥0.9986),检出限和定量限分别为0.30~1.52 ng/L和1.00~5.00 ng/L。在低(8 ng/L)、中(80 ng/L)、高(800 ng/L)3个水平下进行精密度试验,日内和日间精密度(以RSD计)分别为6.5%~17.1%和7.4%~19.4%。该方法应用于地表水、海水、垃圾渗滤液的检测中,检出量为0.6~19.3 ng/L;在8、80、800 ng/L3个水平下进行加标回收试验,回收率为61.7%~120.3%。该方法具有良好的灵敏度、精密度和准确度,为环境水体中苯氧羧酸类物质的痕量检测提供了一种新的检测方法。

## Abstract (en)

Phenoxy carboxylic acid herbicides (PCAs) are difficult to degrade and, thus, pose significant threats to the environment and human health. The limit for 2,4-dichlorophenoxyacetic acid is 30 μg/L in China’s standards for drinking water quality, 70 μg/L in the United States’ drinking water standards, and 30 μg/L in the World Health Organization’s guidelines for drinking water quality. Therefore, the development of an effective detection method for trace PCAs in water is a crucial endeavor. Metal-organic frameworks (MOFs) are novel porous materials that possess advantages such as a large specific surface area, adjustable pore size, and abundant active sites. They exhibit excellent adsorption capability for various compounds. However, the applications of MOFs as adsorbents are limited. For example, the process of isolating powdered MOFs from aqueous solutions is laborious, and microporous MOFs exhibit limited surface affinity, which decreases their mass transfer efficiency in the liquid phase. MOF crystals can be embedded in a substrate to overcome these limitations. Aerogels are obtained by drying hydrogels, which are hydrophilic polymers with a three-dimensional crosslinked network structure. Spongy aerogel materials exhibit unique structural properties such as high porosity, large pore volume, ultralow density, and easy tailorability. When MOFs are combined with an aerogel, their efficient and selective adsorption properties are preserved. In addition, MOF aerogels exhibit a hierarchical porous structure, which enhances the affinity and mass transfer efficiency of the MOF for target molecules. At present, MOF aerogels are primarily prepared by freeze-drying or using supercritical carbon dioxide. These drying processes require significant amounts of energy and time. Hence, the development of greener and more efficient methods to prepare skeleton aerogels is urgently needed. In this study, we prepared an environment-friendly aerogel at ambient temperature and pressure without the use of specialized drying equipment. This ambient-dried MOF composite aerogel was then used for the dispersive solid phase extraction (DSPE) of seven PCAs from environmental water, followed by ultra-high performance liquid chromatography-tandem mass spectrometry (UHPLC-MS/MS). The key parameters affecting the efficiency of DSPE, including the extraction conditions, ratio of MIL-101(Fe)-NH_2_ to sodium alginate, pH of the aqueous samples, extraction time, ionic strength (salinity), and elution conditions, such as the elution solvent ratio, elution time, and elution volume, were investigated to obtain optimal extraction efficiency. The adsorbent could adsorb the target contaminants within 12 min, and the analytes could be completely desorbed within 30 s by elution with 4 mL of 1.5% (v/v) formic acid in methanol solution. The water samples could be analyzed without pH adjustment. The main adsorption mechanisms were electrostatic interactions and *π-π* conjugation. Thus, a new method based on MOF aerogels coupled with UHPLC-MS/MS was developed for the determination of the seven PCA residues in water. The calibration curves for the seven PCAs showed good linearity (*r*^2^≥0.9986), with limits of detection (LODs) and quantification (LOQs) ranging from 0.30 to 1.52 ng/L and from 1.00 to 5.00 ng/L, respectively. Good intra- and inter-day precision values of 6.5%-17.1% and 7.4%-19.4%, respectively, were achieved under low (8 ng/L), medium (80 ng/L), and high (800 ng/L) spiking levels. The developed method was applied to the detection of PCAs in surface water, seawater, and waste leachate, and the detected mass concentrations ranged from 0.6 to 19.3 ng/L. Spiked recovery experiments were conducted at mass concentrations of 8, 80, and 800 ng/L, and the recoveries ranged from 61.7% to 120.3%. The proposed method demonstrates good sensitivity, precision, and accuracy, and has potential applications in the detection of trace PCAs in environmental water.

在农业生产中,苯氧羧酸类除草剂(phenoxy carboxylic acid herbicides, PCAs)是一类使用历史悠久的有机选择性除草剂,被广泛应用于去除作物中的杂草,其水溶性强,极性大,难降解,极易通过地表径流进入到地表水中^[1]^。人体长期接触这类物质,容易罹患低血压、肌无力、发热等疾病,肾脏及肝脏也有可能会受到损伤^[2]^。我国《生活饮用水卫生标准》中规定2,4-滴的限值为30 μg/L, 《美国饮用水水质标准》中的限值为70 μg/L,世界卫生组织颁布的《饮用水水质指南》中的限值为30 μg/L。目前高效液相色谱(HPLC)结合多种检测器是检测PCAs最常用的检测技术,包括HPLC-紫外检测法^[3]^、HPLC-荧光检测法^[4]^、HPLC-二极管阵列检测器法^[5]^、超高效液相色谱-串联质谱法(UHPLC-MS/MS)^[6,7]^。UHPLC-MS/MS相较于HPLC联合多种检测器具有更好的分离效果和灵敏度。实际水样成分复杂,导致分析物的共流出组分影响电喷雾接口的离子化效率,且水中仅含有痕量PCAs,因此需要对水样进行预处理,提高检测的灵敏度。

常用的预处理方法包括固相萃取(SPE)^[8]^、磁固相萃取(MSPE)^[2]^、液液萃取(LLE)^[9]^和固相微萃取(SPME)^[10]^。其中最常用的技术为SPE,具有操作简单,可以对目标物进行高效富集等优点,但传统的商用固相萃取柱存在耗时长、选择性有限等问题,应用受到限制。分散固相萃取(dispersive solid phase extraction, DSPE)不仅具有SPE的优点,而且吸附剂和样品溶液的接触面积大,萃取效率高^[11]^。金属有机骨架材料(metal-organic frameworks, MOFs)作为一种新型的多功能材料,具有比表面积大、孔隙可调的特点。因此,MOFs可作为萃取吸附剂应用于污染物的分析检测。但合成的MOF粉末通常不易分离,且MOF材料大多为微孔结构,这导致MOF材料的亲水性较差,表面传质效率较低,限制了其在水环境中的应用^[12]^。

气凝胶是通过冷冻干燥水凝胶这种具有三维交联网络结构的亲水高分子材料得到的。海绵体的气凝胶材料展现出了一些独特的结构特性,如高孔隙度和大孔体积、超低的密度、柔软易裁剪等。然而气凝胶的多孔结构并不具备良好的选择吸附性。将MOFs与气凝胶相结合,块状MOFs气凝胶可以简化固液分离过程,保留MOFs的高效选择吸附特性。具有多级孔径的MOFs气凝胶可以增强MOFs与目标物分子的亲和性从而具有更快的传质效率。此外,MOFs气凝胶相较于其他气凝胶材料表现出更好的机械强度。近年来,纤维素^[13]^、海藻酸盐^[14]^、卡拉胶^[15]^、壳聚糖^[16]^等高分子材料作为气凝胶在污染物吸附中得到了很好的应用。该类复合气凝胶具有天然、可生物降解、成本低的特点。Bo等^[17]^将ZIF-8与纤维素气凝胶结合用于吸附水中的重金属离子。Zhang等^[18]^采用改进的双冰模板组装方法通过ZIF-67、苯醌改性氧化石墨烯(QGO)和磺基甜菜碱改性壳聚糖(SB)制备了ZIF-67/QGO/SB-CS气凝胶,用于吸附和去除水中的药品和个人护理品。在气凝胶的构建过程中,一个重要的挑战是抵抗界面相互作用引起的崩塌,目前制备气凝胶多采用冷冻干燥方法或使用超临界流体二氧化碳,这些干燥过程需要消耗大量的能量和时间,因此亟需开发出更加绿色环保并且高效的制备MOFs气凝胶的方法。本工作采用MOF材料(MIL-101(Fe)-NH_2_)、海藻酸钠(sodium alginate, SA)作为气凝胶基底,通过常温干燥法制备了MIL-101(Fe)-NH_2_/SA气凝胶,制备过程中产生的CO_2_可以降低毛细管压力和提高海藻酸盐框架的孔隙度。之后,将MIL-101(Fe)-NH_2_/SA气凝胶作为吸附剂,采用DSPE结合UHPLC-MS/MS对水中7种PCAs进行了检测。

## 1 实验部分

### 1.1 仪器、试剂与材料

Triple Quad3500超高效液相色谱-串联质谱仪(美国AB Sciex公司),傅里叶变换红外光谱仪(美国Frontier公司), Sigma300电子扫描显微镜(德国Zeiss公司), ND200氮吹仪(杭州瑞诚仪器有限公司), Millipore D-24UV超纯水机(美国Millipore公司),涡旋混匀仪(上海沪析有限公司)。D8 discover多功能射线X衍射仪(德国Bruker公司), ASAP 2460比表面孔径分析仪(美国Micromeritics公司)。

苯氧羧酸类除草剂标准品:2,4,5-三氯苯氧乙酸(2,4,5-T,纯度>97%)、4-苯氧丁酸(PB,纯度>99%)和2,4,5-三氯苯氧丙酸(2,4,5-TP,纯度>98%)购自德国Dr. Ehrenstorfer公司;4-(2,4-二氯苯氧)丁酸(2,4-DB,纯度≥98%)、2-(2-甲基-4-氯苯氧基)-丙酸(MCPP,纯度>98%)、2-甲基-4-氯苯氧乙酸(MCPA,纯度>98%)、2,4-二氯苯氧乙酸(2,4-D,纯度≥99.8%)和2-氨基对苯二甲酸(2-aminoterephthalic acid, 2-ATA,纯度>98%)购自上海阿拉丁化学试剂有限公司。色谱级甲醇、乙腈购自德国默克公司。SA(1.0~1.2 Pa·s)购自麦克林公司。色谱级甲酸购自天津科密欧化学试剂有限公司。丙酮、碳酸钠、六水合三氯化铁、*N*,*N*-二甲基甲酰胺(*N*,*N*-dimethylformamide, DMF)和乙酸均为分析纯,购自国药集团化学试剂有限公司。氯化钙(纯度>96%)购自天津博迪化工股份有限公司。乙醇(分析纯)购自天津市富宇精细化工有限公司。

实验所用地表水、海水采集于青岛市,垃圾渗滤液采集于青岛市垃圾填埋厂;水样经过0.45 μm水相滤膜过滤后,在4 ℃下储存在棕色瓶中。

### 1.2 标准溶液的配制

分别称取7种PCAs标准品10 mg(精确至0.1 mg),用乙腈溶解并定容至10 mL,配制的质量浓度为1000 mg/L的标准储备液置于棕色小瓶中,于4 ℃下储藏。移取500 μL各标准储备液,用乙腈稀释定容至5 mL,配制的质量浓度为100 mg/L的7种PCAs混合标准工作液置于棕色小瓶中,于4 ℃下保存。临用前用乙腈配制为1 mg/L的混合标准使用液,再用乙腈稀释,配制为所需浓度。

### 1.3 材料制备

#### 1.3.1 制备MIL-101(Fe)-NH_2_

将2.45 mmol的FeCl_3_·6H_2_O和1.24 mmol的2-ATA加入15 mL DMF中,超声30 min混匀。将混合溶液加入到20 mL反应釜中,放入真空烘箱,在110 ℃条件下反应20 h。取出后冷却至室温,4000 r/min下离心10 min。然后用DMF和乙醇依次各清洗3遍。干燥过夜。

#### 1.3.2 制备MIL-101(Fe)-NH_2_/SA气凝胶

如图1所示,合成气凝胶需要经过3个步骤:溶胶凝胶、溶剂交换和室温干燥。

**图1:**
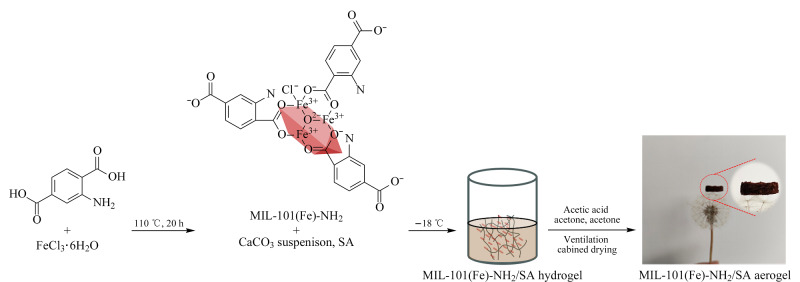
MIL-101(Fe)-NH_2_/SA气凝胶的制备过程

首先将0.58 mmol的SA溶于12.5 mL的超纯水中,超声混匀,静置消泡。随后将1.56 mmol碳酸钠与1.69 mmol的氯化钙分别溶于6.25 mL超纯水中,制得的碳酸钠溶液均匀滴加到氯化钙溶液中,涡旋30 min制为CaCO_3_悬浮液。然后将35 mg MIL-101(Fe)-NH_2_粉末与0.75 mL 碳酸钙悬浮液混合摇匀。摇匀后的液体与2.5 mL的SA溶液混合,移入到5 mL烧杯中。-18 ℃冷冻48 h,得到MIL-101(Fe)-NH_2_水凝胶。

从冰箱中取出,放至室温后,用2 mL 3%乙酸丙酮清洗数遍,乙酸溶解材料中的碳酸钙产生Ca^2+^和CO_2_,使Ca^2+^与SA进一步交联,丙酮置换水凝胶中的水,再用2 mL丙酮清洗数遍去除其中的乙酸,置于通风橱中干燥得到MIL-101(Fe)-NH_2_/SA气凝胶。

### 1.4 样品前处理

将MIL-101(Fe)-NH_2_/SA气凝胶置于50 mL离心管中,向其中加入20 mL水样,调节NaCl浓度为5 mmol/L,用涡旋混匀仪涡旋12 min,使用镊子将材料从水样中取出。然后用4 mL含1.5%甲酸的甲醇洗脱30 s,收集洗脱液,用氮吹仪氮吹至近干。然后用0.5 mL甲醇复溶,经0.22 μm尼龙滤膜过滤后进行UHPLC-MS/MS检测。

### 1.5 仪器条件

色谱条件:ACQUITY UPLC BEH C18色谱柱(100 mm×2.1 mm, 1.7 μm);柱温为40 ℃;流动相为(A)0.01%甲酸水溶液和(B)乙腈;流速为0.4 mL/min。梯度洗脱程序如下^[6]^: 0~1 min, 80%A; 1~3 min, 80%A~55%A; 3~4 min, 55%A; 4~8 min, 55%A~50%A; 8~10 min, 50%A~20%A; 10~11 min, 20%A; 11~11.1 min, 20%A~80%A; 11.1~13 min, 80%A。进样量为5 μL。

质谱条件:电喷雾离子源(ESI源),负离子模式;离子化温度400 ℃;电源电压-4500 V;气帘气压力2.07×10^5^ Pa;雾化气压力3.45×10^5^ Pa;辅助器压力4.14×10^5^ Pa;多反应监测模式。7种PCAs的其他质谱参数见表1。

**表1:** 7种PCAs的质谱参数

Analyte	Abbreviation	*t*_R_/ min	Precursor ions (*m/z*)	Product ions (*m/z*)	Declustering potentials/V	Collision energies/eV
4-Phenoxybutyric acid	PB	3.92	178.0^*^	93.0	-54	-24
(4-苯氧丁酸)						
5-Trichlorophenoxyacetic acid	2,4,5-T	5.09	252.7^*^, 254.9	194.8, 196.9	-45, -44	-20, -22
(2,4,5-三氯苯氧乙酸)						
2-(2,4,5-Trichlorophenoxy) propanoic acid	2,4,5-TP	6.25	268.7^*^, 266.8	196.9, 195.0	-45, -48	-25, -16
(2,4,5-三氯苯氧丙酸)						
2,4-Dichlorophenoxybutyric acid	2,4-DB	6.02	247.0^*^, 249.0	161.0, 163.0	-40, -40	-18, -18
(4-(2,4-二氯苯氧)丁酸)						
2-Methyl-4-chlorophenoxyacetic acid	MCPA	4.39	199.0^*^, 201.0	141.0, 143.0	-45, -45	-18, -13
(2-甲基-4-氯苯氧乙酸)						
2-Methyl-4-chlorophenoxypropionic acid	MCPP	5.19	213.0^*^, 215.0	141.0, 143.0	-50, -50	-22, -22
(2-(2-甲基-4-氯苯氧基)-丙酸)						
2,4-Dichlorophenoxyacetic acid	2,4-D	4.27	219.0^*^, 221.0	161.0, 163.0	-37, -37	-18, -18
(2,4-二氯苯氧乙酸)						

* Quantitative ion.

## 2 结果与讨论

### 2.1 材料的表征

图2为MIL-101(Fe)-NH_2_/SA气凝胶和MIL-101(Fe)-NH_2_的红外光谱图。如图2a示,3466 cm^-1^处的峰对应N-H的伸缩振动,1259 cm^-1^处的峰对应C-N的伸缩振动,表明MIL-101(Fe)-NH_2_中氨基的存在;1424 cm^-1^和1704 cm^-1^处的峰分别对应MIL-101(Fe)-NH_2_中C-O和C=O的伸缩振动,表明羧基的存在;1495 cm^-1^和1582 cm^-1^处的峰对应MIL-101(Fe)-NH_2_中苯环的伸缩振动;441 cm^-1^处的峰对应MIL-101(Fe)-NH_2_中Fe-O的伸缩振动;以上红外光谱结果表明MIL-101(Fe)-NH_2_成功合成。图2b中3324 cm^-1^附近的峰对应-OH的伸缩振动;1664 cm^-1^处的峰对应C=O的伸缩振动;1065 cm^-1^处的峰对应SA中C-O-C的对称伸缩振动;并且MIL-101(Fe)-NH_2_/SA气凝胶中也含有MIL-101(Fe)-NH_2_的相关特征峰,这说明制备的复合气凝胶没有损坏MIL-101(Fe)-NH_2_的结构。

**图2:**
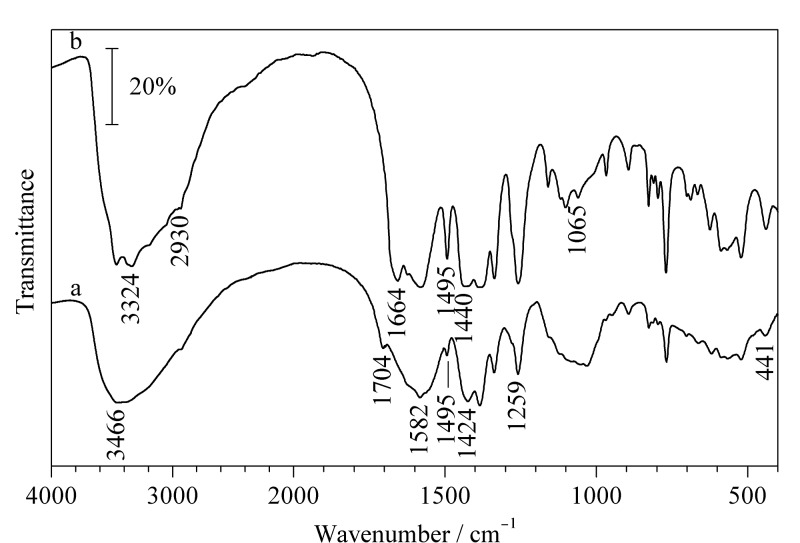
(a)MIL-101(Fe)-NH_2_和(b) MIL-101(Fe)-NH_2_/SA气凝胶的红外光谱图

图3为MIL-101(Fe)-NH_2_/SA气凝胶和MIL-101(Fe)-NH_2_的扫描电镜图。如图3a~c所示,制备的MIL-101(Fe)-NH_2_/SA气凝胶整体为层状多孔结构,且MIL-101(Fe)-NH_2_颗粒均匀分布在气凝胶表面。如图3d所示,MIL-101(Fe)-NH_2_具有良好的分散性,晶体长度约为500 nm。

**图3:**
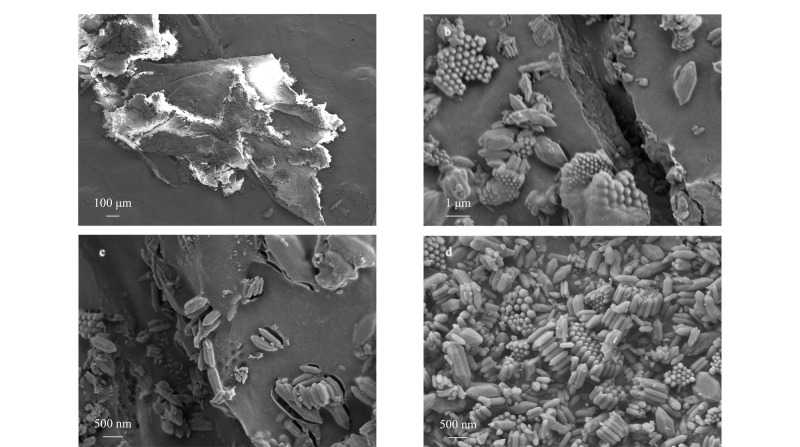
(a、b、c)MIL-101(Fe)-NH_2_/SA气凝胶和(d)MIL-101(Fe)-NH_2_的扫描电镜图

图4是MIL-101(Fe)-NH_2_/SA和MIL-101(Fe)-NH_2_的N_2_吸附-解吸等温线,MIL-101(Fe)-NH_2_/SA气凝胶的比表面积为100.83 m^2^/g, MIL-101(Fe)-NH_2_的比表面积为162.42 m^2^/g。由图4可知,MIL-101(Fe)-NH_2_/SA气凝胶具有更小的比表面积,MIL-101(Fe)-NH_2_/SA气凝胶存在轻微的滞后环,表明存在介孔结构。这说明MIL-101(Fe)-NH_2_/SA气凝胶既有MOF的微孔结构又有介孔结构,具有分级多孔结构,传质速率快。

**图4:**
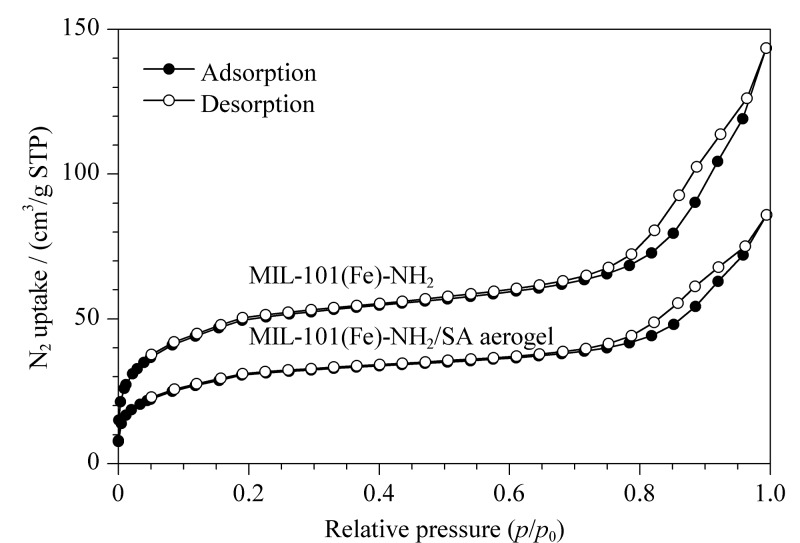
MIL-101(Fe)-NH_2_/SA气凝胶和MIL-101(Fe)-NH_2_ 的N_2_吸附-解吸等温线图

如图5a所示,MIL-101(Fe)-NH_2_的典型峰为9.58°、10.72°、16.84°、18.92°,与文献[19]中的典型峰位置相似。MIL-101(Fe)-NH_2_/SA气凝胶具有与MIL-101(Fe)-NH_2_相似的特征峰位,这说明在气凝胶的合成过程中,MIL-101(Fe)-NH_2_的结晶结构没有发生改变。

**图5:**
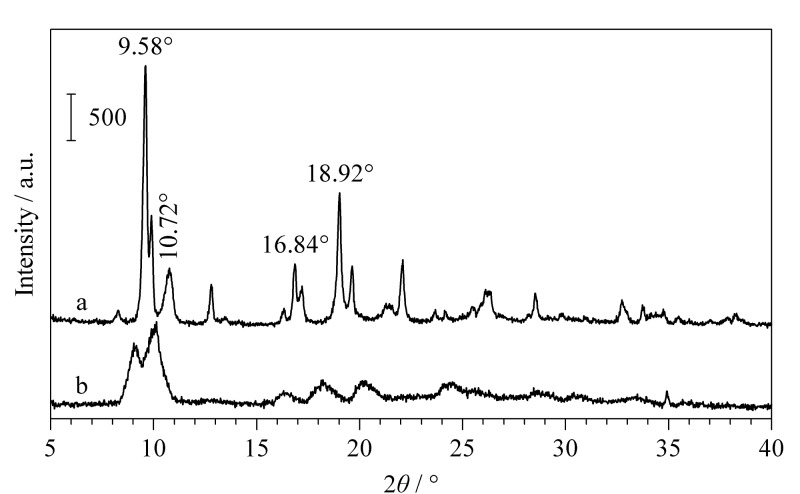
(a)MIL-101(Fe)-NH_2_和(b)MIL-101(Fe)-NH_2_/SA气凝胶的XRD图谱

### 2.2 前处理条件的优化

#### 2.2.1 气凝胶中MIL-101(Fe)-NH_2_与SA的比例

实验分别考察了MIL-101(Fe)-NH_2_与SA的质量比为0∶1、0.5∶1、0.8∶1、1∶1、1.2∶1、1.4∶1、1.6∶1时PCAs的萃取效果。如图6a所示,当MOF与SA的质量比为1.4∶1时萃取效果最好,随后增加MOF所占比例,萃取效率略有下降。这可能是由于MOF量过多时材料易碎,从而使萃取效率下降。

**图6:**
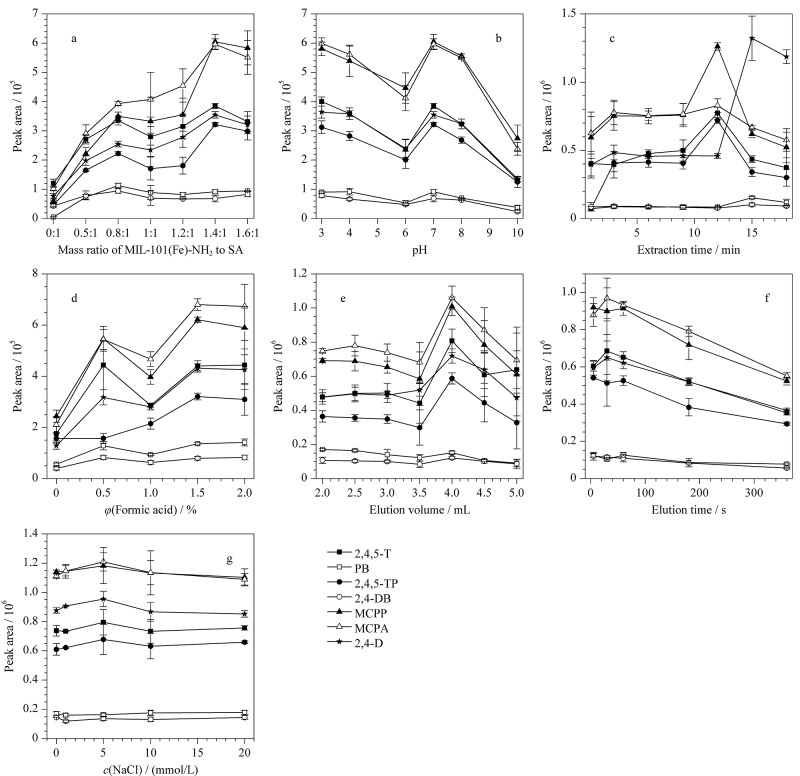
(a) MIL-101(Fe)-NH_2_与SA的质量比、(b)水样pH、(c)萃取时间、(d)洗脱溶剂甲醇中甲酸体积分数、(e)洗脱体积、(f)洗脱时间、(g)盐度对7种PCAs萃取效率的影响(*n*=3)

#### 2.2.2 水样pH值

实验考察了水样pH为3、4、6、7、8、10时PCAs的萃取效果。实验结果如图6b所示,随着水样pH值的增加,pH在3~6时萃取效率下降,pH在6~7时上升,随后下降。这是由于这7种PCAs的p*K*_a_均在2.64~4.58范围内,当水样pH为3~6时,PCAs中的羧酸基团逐渐解离,由中性分子逐渐变为阴离子,PCAs与MOF材料的*π*-*π*作用力减弱,导致萃取效率降低。pH=7时,PCAs完全离子化,此时PCAs带负电,MIL-101(Fe)-NH_2_带正电,MIL-101(Fe)-NH_2_上氨基与PCAs中羧基之间的静电作用占主导作用,萃取效率达到最佳。当pH>8时,MOF表面携带大量阴离子,与目标物所带负电产生静电斥力导致萃取效率下降。因此后续实验选择不调节pH值(pH值约为7)。

#### 2.2.3 萃取时间

不同的萃取时间会影响MIL-101(Fe)-NH_2_/SA气凝胶对PCAs的吸附效果。如图6c所示,有4种除草剂在萃取时间为12 min时吸附洗脱效果最好,有两种物质在15 min时吸附洗脱效果最好,但在15 min时有4种除草剂的吸附洗脱效果出现明显降低,因此选择12 min为萃取时间。

#### 2.2.4 洗脱条件

洗脱条件主要考察了洗脱溶剂中甲酸和甲醇的比例、洗脱体积以及洗脱时间。甲醇作为一种极性较强的溶剂,可以对PCAs进行有效洗脱,甲酸可以破坏氢键从而使PCAs更容易从凝胶上脱离。因此采用含0、0.5%、1%、1.5%、2%甲酸的甲醇对PCAs进行洗脱。如图6d所示,甲酸体积分数为1.5%时,PCAs的洗脱效果最好,增加甲酸比例,洗脱效果无明显变化,因此选择洗脱溶剂甲醇中甲酸的体积分数为1.5%。考察了洗脱体积为2.0、2.5、3.0、3.5、4.0、4.5、5.0 mL、单次洗脱条件下的洗脱效果。如图6e所示,洗脱体积为4 mL时,洗脱效果最好。因此选择4 mL为最佳洗脱体积。洗脱时间影响目标物从吸附材料上的脱附效果,考察洗脱时间为5、30、60、180、360 s时PCAs的洗脱效果。如图6f所示,洗脱时间为30 s时,洗脱溶剂对目标物的洗脱效果最好,因此选择洗脱时间为30 s。

#### 2.2.5 离子强度

考察了NaCl浓度为0、1、5、10、20 mmol/L时的吸附洗脱效果。由于盐析效应,水分子会在NaCl分子周围形成水合球,从而降低分析物的溶解度,有助于增强目标分析物向固体材料移动,增强富集作用。如图6g所示,添加NaCl浓度为5 mmol/L时吸附洗脱效果最好,而随着NaCl浓度的进一步增加,会使水样的黏度进一步增强从而减缓其传质效率。因此选择调节NaCl浓度为5 mmol/L。

### 2.3 基质效应(ME)

将地表水、海水、渗滤液的空白基质液作为溶剂配制50 μg/L的基质标准溶液,用乙腈作为溶剂配制50 μg/L的纯溶剂标准溶液。使用公式ME=*A/B*^[20]^计算基质效应,其中*A*为基质匹配标准溶液中目标物的峰面积,*B*为纯溶剂标准溶液中目标物的峰面积。0.8≤ME≤1.2表示基质效应不明显;0.5≤ME<0.8以及1.2<ME≤1.5表示中等基质效应;ME<0.5以及ME>1.5表示较强基质效应^[21]^。如表2所示,7种PCAs的基质效应为低、中基质效应,可以通过基质匹配曲线进行基质校正^[22]^,因此本工作采用基质匹配曲线进行定量分析。

**表2:** 7种PCAs的基质效应

No.	Compound	MEs		
		Seawater	Surface water	Leachate
1	2,4,5-T	1.45	1.40	1.35
2	PB	0.66	0.79	0.70
3	2,4,5-TP	1.46	1.37	1.37
4	2,4-DB	0.93	1.08	0.89
5	MCPA	1.25	1.27	1.22
6	MCPP	1.18	1.33	1.66
7	2,4-D	0.52	1.36	0.50

### 2.4 方法分析性能

#### 2.4.1 线性范围、检出限和定量限

在优化的萃取条件下,考察了建立的DSPE-UHPLC-MS/MS分析方法的性能。将7种PCAs的混合标准使用液用空白基质液稀释至浓度梯度为1、5、8、50、80、500、800、1000 ng/L,作为模拟水样,经过样品前处理,建立7种PCAs的基质匹配标准曲线。如表3所示,2,4-DB在5.0~1000 ng/L范围内具有良好的线性关系;其余6种PCA在1.0~1000 ng/L范围内具有良好的线性关系。它们的线性相关系数(*r*^2^)为0.9986~0.9997,检出限(LOD)和定量限(LOQ)分别通过3倍和10倍信噪比(*S/N*)获得。LOD和LOQ分别为0.30~1.52 ng/L和1.00~5.00 ng/L。

**表3:** 7种PCAs的线性方程、相关系数、线性范围、检出限和定量限

Analyte	Linear equation	*r*^2^	Linear range/(ng/L)	LOD/(ng/L)	LOQ/(ng/L)
2,4,5-T	*y*=647.16*x*+2825.3	0.9988	1.0-1000	0.30	1.00
PB	*y*=246.59*x*+271.0	0.9959	1.0-1000	0.30	1.00
2,4,5-TP	*y*=584.49*x*+3879.7	0.9995	1.0-1000	0.30	1.00
2,4-DB	*y*=125.82*x*+646.6	0.9997	5.0-1000	1.52	5.00
MCPP	*y*=845.25*x*+2306.6	0.9997	1.0-1000	0.30	1.00
MCPA	*y*=738.90*x*+4306.1	0.9986	1.0-1000	0.30	1.00
2,4-D	*y*=582.53*x*+4108.0	0.9990	1.0-1000	0.30	1.00

*y*: peak area; *x*: mass concentration, ng/L.

#### 2.4.2 精密度

将7种PCAs混合标准储备液用超纯水稀释为低(8 ng/L)、中(80 ng/L)、高(800 ng/L)3个水平,进行加标回收试验。在1天中对3个加标水平的5个水平样品进行测定,计算日内精密度(RSD);连续测定5天,计算日间精密度(RSD)。以上实验用于考察检测方法的重复性。如表4所示,日内和日间精密度分别为6.5%~17.1%和7.4%~19.4%, 表明建立的基于MIL-101(Fe)-NH_2_/SA气凝胶的DSPE-UHPLC-MS/MS方法用于测定水中7种PCAs具有良好的精密度。

**表4:** 7种PCAs的日内和日间精密度(*n*=5)

Analyte	Spiked/ (ng/L)	RSDs/%	
		Intra-day	Inter-day
2,4,5-T	8	9.8	12.6
	80	9.0	14.1
	800	8.2	11.9
PB	8	14.2	14.8
	80	11.0	12.6
	800	8.6	8.2
2,4,5-TP	8	14.3	14.2
	80	7.6	19.4
	800	9.7	10.8
2,4-DB	8	17.1	18.1
	80	9.2	14.0
	800	10.9	17.1
MCPP	8	10.9	8.2
	80	8.5	18.6
	800	8.1	7.4
MCPA	8	6.9	17.2
	80	9.6	10.3
	800	9.1	8.2
2,4-D	8	14.9	10.8
	80	8.1	12.9
	800	6.5	12.7

### 2.5 实际样品分析及回收率测定

对地表水、海水以及垃圾渗滤液实际样品进行分析,结果如表5所示,地表水中7种PCAs都有检出,在海水中检测到痕量的MCPA和2,4-D,在垃圾渗滤液中检测到痕量的5种PCAs。在3种不同水样中进行低、中、高3个水平的加标回收试验,加标回收率为61.7%~120.3%。

**表5:** 实际水样中7种苯氧羧酸类除草剂的含量、加标回收率及其RSD(*n*=3)

Analyte	Spiked/ (ng/L)	Seawater				Surface water				Leachate		
		Background/ (ng/L)	Recovery/ %	RSD/ %		Background/ (ng/L)	Recovery/ %	RSD/ %		Background/ (ng/L)	Recovery/ %	RSD/ %
2,4,5-T	0	ND				4.6				0.6		
	8	8.3	103.4	12.7		14.1	112.2	9.6		6.8	85.2	12.7
	80	73.7	92.2	11.6		98.7	116.7	12.5		82.9	103.7	11.6
	800	766.7	95.8	20.4		806.3	100.2	11.1		754.8	94.4	20.4
PB	0	ND				14.2				3.6		
	8	8.6	107.1	8.6		17.7	79.6	10.0		13.6	117.3	8.6
	80	85.6	107.0	11.6		73.4	78.0	12.2		70.1	87.6	11.6
	800	718.4	89.8	15.0		788.0	96.8	12.2		775.2	96.9	15.0
2,4,5-TP	0	ND				3.6				ND		
	8	9.3	116.8	10.8		14.0	120.3	12.8		7.9	99.4	10.8
	80	83.7	104.6	2.0		87.6	104.8	18.7		85.2	106.5	2.0
	800	810.7	101.3	1.9		723.7	90.1	13.1		756.9	94.6	1.9
2,4-DB	0	ND				3.1				8.2		
	8	9.5	118.7	16.4		8.8	79.4	6.8		9.9	61.7	16.4
	80	78.3	97.9	19.4		83.3	100.4	3.5		76.2	86.6	19.4
	800	827.4	103.4	4.6		782.0	97.4	19.4		728.2	90.1	4.6
MCPP	0	ND				9.8				ND		
	8	6.5	81.5	5.4		20.4	114.7	4.0		5.1	64.0	5.4
	80	73.0	91.0	10.7		77.1	85.8	18.3		83.5	104.4	10.7
	800	774.5	96.8	4.7		714.1	88.2	10.6		769.7	96.2	4.7
MCPA	0	0.6				19.3				4.7		
	8	7.4	86.5	10.7		30.3	111.0	11.0		9.1	63.7	10.7
	80	67.1	83.2	17.9		98.4	99.1	11.8		80.3	100.4	17.9
	800	806.8	100.8	1.1		806.2	98.4	11.1		799.5	99.9	1.1
2,4-D	0	0.8				4.5				4.0		
	8	8.1	91.7	20.2		15.0	119.4	1.4		6.4	79.5	20.1
	80	67.8	83.9	17.2		92.8	109.8	19.1		69.7	87.2	17.2
	800	724.2	90.4	20.1		748.6	93.0	6.9		587.7	73.5	18.9

### 2.6 与文献方法对比

将所建立方法与文献中检测苯氧羧酸类物质的方法相比较(见表6),可以看出,本文方法具有较低的检出限及较快的萃取速度。此外,本文所使用常温干燥法制备的材料绿色、环保,具有更快的洗脱时间(30 s),并且拓展了在垃圾渗滤液中检测苯氧羧酸类物质的应用。

**表6:** 本方法与其他文献中PCAs检测方法的比较

Material	Method	Matrices	Extraction time/min	LOD/ (ng/L)	Ref.
TAPA-TFPB-COFs	SPE-HPLC-MS/MS	tap water, well water, river water	>120	0.30-0.56	[8]
NH_2_-MWCNTs	MSPE-UHPLC-MS/MS	lake water, river water, farmland water, tap water	10	10.00-20.00	[23]
CoFe_2_O_4_/PCPS	MSPE-HPLC-UV	river water, lake water and snow water	10	300.00-590.00	[3]
GEM/MMF-SPME	MMF/SPME-HPLC-DAD	rice	30	360.00-660.00	[24]
MMIP	HPLC-DAD	mineral, tap, fountain, well	30	330.00-710.00	[5]
MIL-101(Fe)-NH_2_/SA	DSPE-UHPLC-MS/MS	seawater, surface water, leachate	12	0.30-1.52	this work

MSPE: magnetic solid phase extraction; MMF: multiply monolithic fiber.

## 3 结论

本文通过常温干燥法制备的MIL-101(Fe)-NH_2_/SA气凝胶不仅可以有效地选择性吸附PCAs,环保,经济,还可以更好地实现固液分离。结合DSPE与UHPLC-MS/MS检测水中7种PCAs,方法的灵敏度和准确度可以满足实际水样的检测需求。该方法成功应用于地表水、海水和垃圾渗滤液的检测中,为苯氧羧酸类物质在水样中的痕量检测提供了新方法、新思路。

## References

[1] Huang Y F, Liu J, Huang X J. Chinese Journal of Chromatography, 2022, 40(10): 900 10.3724/SP.J.1123.2021.12008 PMC9577702 36222253

[2] Gurav R, Mandal S, Smith L M, et al. Chemosphere, 2023, 339: 139715 37536539 10.1016/j.chemosphere.2023.139715

[3] Guo X L, Li Y T, Zhang B, et al. Microchem J, 2020, 152: 104443

[4] Yu B C, Zhen L, Wu J Y. Anal Lett, 2020, 53(5): 746

[5] Meseguer-Lloret S, Torres-Cartas S, Gómez-Benito C, et al. Talanta, 2022, 239(1): 123082 10.1016/j.talanta.2021.123082 34823860

[6] Ji X F, Li S, Wu G G, et al. Chinese Journal of Chromatography, 2021, 39(8): 896 10.3724/SP.J.1123.2021.01006 PMC9404032 34212590

[7] Zhang X, Han L X, Qiu T, et al. Chinese Journal of Chromatography, 2023, 41(3): 224 10.3724/SP.J.1123.2022.05005 PMC9982704 36861205

[8] Geng H S, Xu G J, Liu L, et al. J Chromatogr A, 2022, 1682: 463516 36162252 10.1016/j.chroma.2022.463516

[9] Tran A T, Hyne R V, Doble P. Chemosphere, 2007, 67(5): 944 10.1016/j.chemosphere.2006.11.002 17184816

[10] Wu J Y, Chen H X, Huang X J. J Chromatogr A, 2021, 1653: 462407 34315063 10.1016/j.chroma.2021.462407

[11] Ge F, Li M M, Ye H. J Hazard Mater, 2012, 211: 366 22209322 10.1016/j.jhazmat.2011.12.013

[12] Wan L Y, Xu H, Gao J K, et al. Coor Chem Rev, 2019, 398(1): 213016

[13] Lei C, Gao J K, Ren W J, et al. Carbohyd Polym, 2019, 205: 35 10.1016/j.carbpol.2018.10.029 30446114

[14] Semin E, Ryu J H, Kim H C. J Environ Manage, 2021, 297: 113389 34325366 10.1016/j.jenvman.2021.113389

[15] Song G, Shi Y, Li A, et al. J Mater Sci, 2021, 56: 14866

[16] Ling H J, Wu G G, Li S, et al. Chinese Journal of Chromatography, 2022, 40(4): 323 10.3724/SP.J.1123.2021.07014 PMC9404051 35362680

[17] Bo S G, Ren W J, Lei C. J Solid State Chem, 2018, 262(1): 1

[18] Zhang W K, Huang T, Ren Y, et al. Carbohyd Polym, 2022, 298(15): 120102 10.1016/j.carbpol.2022.120102 36241325

[19] Zhang H X, Hu X Z, Xia H. Chem Pap, 2022, 76: 4379

[20] Mi K, Zhang W T, Wen L H, et al. Chinese Journal of Chromatography, 2023, 41(12): 1141 10.3724/SP.J.1123.2023.04002 PMC10719801 38093545

[21] Ferrer C, Lozano A, Agueera A, et al. J Chromatogr A, 2011, 1218(42): 7634 10.1016/j.chroma.2011.07.033 21820661

[22] Hu B, Li L X, Ding X P, et al. Chinese Journal of Chromatography, 2024, 42(1): 38 10.3724/SP.J.1123.2023.04012 PMC10782276 38197205

[23] Peng M M, Han Y Q, Xia H, et al. J Sep Sci, 2018, 41(10): 2221 29430822 10.1002/jssc.201701325

[24] Pei M, Shi X L, Wu J Y, et al. Talanta, 2019, 191: 257 30262059 10.1016/j.talanta.2018.08.073

